# Health-related quality of life and its relationship with clinical symptoms among Iranian patients with polycystic ovarian syndrome

**Published:** 2013-05

**Authors:** Fatemeh Bazarganipour, Saeide Ziaei, Ali Montazeri, Fatemeh Foroozanfard, Soghrat Faghihzadeh

**Affiliations:** 1 *Department of Reproductive Health, Faculty of Medical Sciences, Tarbiat Modares University, Tehran, Iran.*; 2 *Mental Health Research Group, Health Metrics Research Center, Iranian Institute for Health Sciences Research, ACECR, Tehran,Iran.*; 3 *Department of Obstetrics and Gynecology, Kashan University of Medical Sciences, Kashan, Iran.*; 4 *Faculty of Medical Sciences, Zanjan University of Medical Sciences, Zanjan, Iran.*

**Keywords:** *Quality of life*, *Polycystic ovarian syndrome*, *Iran*

## Abstract

**Background: **Polycystic ovarian syndrome (PCOS) has been shown to cause a reduction in Health-related quality of life (HRQOL).

**Objective:** This study examines the extent of different clinical symptoms in PCOS patients on HRQOL.

**Materials and Methods: **A cross-sectional study was undertaken to ascertain the factors related to HRQOL in 200 PCOS patients in Kashan, Iran. Main outcome measures were modified polycystic ovarian syndrome questionnaire (MPCOSQ) and clinical information of PCOS. Major clinical PCOS features including obesity (BMI), excessive body hair (hirsutism score), acne, menstrual cycle disturbances and infertility.

**Results:** Findings showed that the most common HRQOL concern was menstrual irregularities and infertility, followed in descending order by hirsutism, weight, emotion, and acne. Multivariate analysis revealed the menstrual irregularities as a significant predictor of menstruation (p=0.005), emotion (p=0.02) and infertility (p=0.02) subscales of the MPCOSQ. Having of infertility, predicted scores on the infertility subscale (p<0.0001). Hirsutism score was a significant predictor of hirsutism (p<0.0001) and emotion (p<0.0001) subscales. Weight subscale concerns was predicted by BMI (p<0.0001), also, acne was found to be predicted score of acne subscale (p<0.0001).

**Conclusion:** Worsened HRQOL in women with PCOS was related to more menstrual irregularities and infertility than to obesity. The finding suggests a potential for poorer compliance with weight management protocols among affected PCOS patients.

## Introduction

Polycystic ovarian syndrome (PCOS) is the most common endocrine disorder in women of reproductive age. It is estimated that 5-10% of women suffer from the disease (1). The symptoms typically associated with PCOS, including amenorrhea, oligomenorrhea, hirsutism, obesity, infertility, anovulation and acne, can lead to mood disturbances including symptoms of depression, marital and social maladjustment and impair sexual functioning (2). 

The research evidence suggests that women with PCOS experience substantial pressure related to their symptoms. Comparing women with PCOS with healthy controls, other gynecological populations and patients with asthma, epilepsy, diabetes, low back pain, arthritis and coronary heart disease, it has been shown that PCOS had a greater impact on women’s psychological wellbeing (2-5). Studies of women with (for any cause) hirsutism, obesity, amenorrhea and infertility (common symptoms of PCOS), show considerable distress and anxiety related to their failure to conform to idealized ‘feminine’ norms of appearance (6-8). 

Reviewing available literature, one can find many studies that confirm symptoms of PCOS causes a major reduction in the Health-related quality of life (HRQOL) of affected women. Hahn *et al*, assessing the quality of life (QOL), psychological well-being and sexual satisfaction of 120 patients with the diagnosed PCOS, showed a subjective deterioration of general well-being, increase of psychological disturbances and sexual problems in women with PCOS. Moreover, the authors defined the risk factors for the above mentioned disturbances in women with PCOS, which included hirsutism and obesity (2). 

Ching *et al* analyzing the QOL parameters of 203 patients with PCOS at the age of 15-65 years in a cross-sectional study (Short Form-36 -SH-36, Polycystic Ovary Syndrome Questionnaire- PCOSQ, General Health Questionnaire- 28- GHQ- 28), showed a significant reduction QOL in women with PCOS in comparison with the healthy women population. In 62.4% of PCOS, there are psychological disturbances (p<0.001). Hirsutism, BMI and menstuaral irregularities negatively correlated with the QOL domains (5). The issue of the QOL of patients with PCOS is very often overlooked in the clinical practice (2). 

As there is no cure for PCOS now, management should be aimed at improving the patients HRQOL by means of symptomatic improvement and prevention of long-standing complications. The value of including health-related quality of life (HRQOL) to evaluate treatment from the patient’s perspective and to investigate clinical interventions over time is well established (9-11). In addition, investigators have identified the need for exploring the impact upon HRQOL in different cultures since cultural-based traditions and gender identity may influence HRQOL of women with PCOS (12, 13).

 The prevalence of PCOS in Iran is relatively high (11.4%) and studying HRQOL in these patients seems important (14). Problems related to HRQOL among non-western women have not been reported in detail; therefore, a study on this topic is necessary. To our best knowledge, this is the first study that reports on the topic. It is seemed this might help to add to the existing knowledge and perhaps improve health in women with PCOS. This study was conducted to examine HRQOL who suffer from the disease.

## Materials and methods

**Design and data collection**

This was a cross-sectional study of women with PCOS who attended two private gynecology clinics in Kashan, Iran from October 2011 to January 2012. The Ethics Committee of the Tarbiat Modares University approved the study. This study was funded by grant of Tarbiat Modares University, Iran. The sample size was determined based on the following formula:

$n=\frac{(z^{2}\times s^{2}}{d^{2}}$

S and d were calculated based on previous studies [mean and standard deviation in each domains of modified polycystic ovarian syndrome questionnaire (MPCOSQ)] and maximum sample size for this study was calculated. By considering, z=1.96, the primary sample size was determined at least 185. All women who had been previously diagnosed with PCOS and were scheduled for a routine follow-up appointment were invited to participate with convenience sampling method. All participants provided written informed consent. 

A confirmed case of PCOS was diagnosed based on the definition of the Rotterdam diagnostic criteria, i.e. having any two of the following: 1) Polycystic ovaries visualized on ultrasound scan (presence of 12 follicles or more in one or both ovaries and/or increased ovarian volume i.e. >10 ml), 2) clinical signs of hyperandrogenism (hirsutism score based on hirsutism score greater than 7 or obvious acne) and/or an elevated plasma testosterone (testosterone >2.0 nmol/l), 3) having an interval between menstrual periods >35 days and/or amenorrhea, defined as the absence of vaginal bleeding for at least 6 months (i.e.199 days).

Patients were eligible if they met each of the following criteria: being 15-40 years of age; married; not having abnormally elevated values of prolactin, thyroid stimulation hormone (TSH), and FSH; Iranian; any problems in speaking or listening; and not having another casue for infertility (for example male factor). Main outcome measures were MPCOSQ and clinical information of PCOS as below:

**Health-related quality of life measure**

The MPCOSQ include 30 questions from six HRQOL areas or domains: emotional disturbances (8 items), hirsutism (5 items), infertility (4 items), weight (5 items), menstrual (4 items) and acne (4 items). Each item is associated with a seven-point Likert scale, in which a score of 7 suggest no problems or difficulties and solve 1 indicates maximum HRQOL impairment on that item (15-16). Psychometric properties of MPCOSQ in Iranian population have been verified (17).

**Clinical outcomes**

Moreover to socio-demographic characterize, several information related to clinical symptoms of PCOS was considered as followed: 

1) Menstrual history: interval between menstruation periods during last 12 months were asked from all patients and categorized to <21 days, 21-34 days, 35-60 days, >199 days and changeable.

2) Infertility history: having infertility and its duration was recorded according to case reports.

3) BMI: weight and height were calculated by weight/ height squared [kg/m^2^] in all patients.

4) Body hair: clinical assessment of hirsutism was determined using the Ferriman-Gallwey Scoring System (F/G score). Based on this scale, nine body sites (the upper lip, chin, chest, upper back, lower back, upper abdomen, lower abdomen, arm, and thigh) were graded from 0 (no terminal hair) to 4 (severe hirsutism). Scores can range from 0 to 36. A score of 7 or above was considered positive for hirsutism (18). 

5) Acne: acne was determined by observation of patients (presence or absence of acne).

**Statistical analysis**

Data are presented as number (%), unless otherwise indicated. The impact of PCOS symptoms on HRQOL was explored by including all explanatory variables listed in Table I. Because the study included many independent variables, an adjusted R^2^ was used. A stepwise approach was used to exclude factors that did not contribute significantly to the HRQL scores. Categorical variables were dichotomized and converted and coded as dummy variables. For example, menstrual cycle was converted to 1= having amenorrhea, oligomenorrhea or changeable; 0= the remaining. 

No multicollinearity problems were encountered when variance inflation factor (VIF) were analyzed. The normal distribution of the standardized residuals was examined graphically by normal probability plots and tested by the Kolmogorov-Smirnov test. Statistical analysis was performed using Statistical Package for the Social Sciences 15.0 (SPSS Inc., Chicago, IL, USA). P<0.05 was accepted as significant.

## Results

**Socio-demographic characterize and clinical symptoms**

During the 6-month enrollment, 320 women were invited to participate; 215 women consented and were enrolled, and 200 women completed surveys. The mean age of patients was 28.02 (SD=6.11) years. The majority of women had education beyond high school (51.5%, n=103). 

Most women were housewife (80%, n=160). The majority of women had normal BMI and approximately one in five of the sample exhibiting a BMI greater than 30. Approximately, half of the women exhibited clinical features of hirsutism; with mean (±SD) F/G scores for the entire PCOS were 8.39±4.28. Two thirds of the sample had infertility and changeable menstruation. Socio-demographic and clinical characteristic of the patients are presented in table I.

**MPCOSQ subscale’s scores**

**Figure 1:**
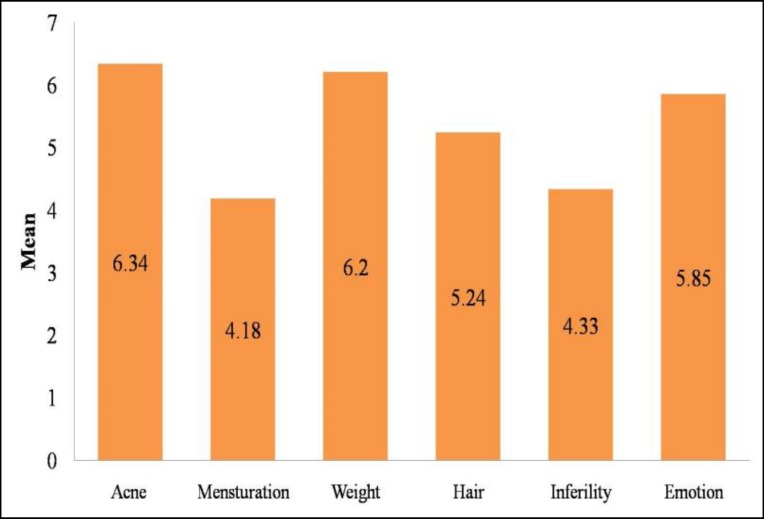
Mean scores of MPCOSQ’s subscales in PCOS patients. 1= poorest functioning or greater concern, 7= optimal functioning or lower concern

Figure 1 presents a summary of the mean scores of the six subscales of the MPCOSQ. In order of importance from greatest to least concern were the scores for (1) menstrual problems, (2) infertility, (3) hirsutism, (4), weight, (5) emotions and finally (6) acne. 

**Factors contributing to MPCOSQ scores according to multiple linear regression**

The patients’ characteristics according to multivariate linear regression were predictive of the subscale of MPCOSQ scores are presented in table II. Menstrual irregularities (amenorrhea, oligomenorrhea and changeable) predicted score of menstruation subscale (=-0.225, p=0.005). Women with PCOS who had irregular menstruation reported the lowest scores (greatest concerns) on the menstrual subscale (indicating poorer functioning) than the other groups. 

Menstrual irregularities was also found to be predicted score of emotions subscale (=-0.496, p=0.02) and infertility (=-0.676, p=0.022) of the MPCOSQ. In the other word, having menstrual irregularities result in the lower of the scores on emotional and infertility subscales of MPCOSQ. Having of infertility, predicted scores on the infertility concern subscale of the MPCOSQ (=-1.631, p<0.0001) that the higher of duration of infertility, the lower the scores on infertility subscale of MPCOSQ. 

The third lowest reported subscale of concern in the overall MPCOSQ was hirsutism. This subscale was predicted by F/G score (=-0.22, p<0.0001). The higher the F/G score, the lower the reported body hair score, which was indicative of poorer functioning. The F/G score was also found to be predicted score of emotions subscale of MPCOSQ (=-8.86, p<0.0001). 

Weight subscale concerns was predicted by BMI (=-0.768, p<0.0001), such that the higher the BMI (more obese), the lower the scores reported on the weight subscale of MPCOSQ. Also, acne was found to be predicted score of acne subscale of the MPCOSQ (=-1.212, p<0.0001).

**Table I:** Socio-demographic and clinical characteristics in PCOS patients

**Variable**		
Age (years)*[Mean ± SD]		28.02 ± 6.11
Education **		
	Guidance school or less	44 (22)
	Completed high school	103 (51.5)
	College	53 (26.5)
Occupation **		
	Employment	39 (19.5)
	Housewife	160 (80)
Hirsutism score**		
	Normal F/G	113 (56.5)
	Hyperandrogenism F/G	87 (43.5)
Having Acne **		121 (60.5)
Interval between menstruation (days)**		
	< 21	48 (24)
	21-34	53 (26.5)
	35-60	19 (9.5)
	>199	12 (6)
	Changeable	68 (34)
Diagnosis of Infertility **		135 (67.5)
BMI (kg/m^2^) **		
	< 25	81 (40.5)
	25-30	87 (43.5)
	> 30	32 (16)

** N (%)                     FG: Ferriman-Gallwey score

**Table II:** Multiple linear regression analysis including socio-demographic and clinical symptoms predictive of HRQOL scores among PCOS patients

**Outcome variable** ^*^	**Independent variables**	**Coefficient ()**	**SE**	**p-value**	**95% CI**	**Adjusted R** ^2^
Hirsutism	F/G score	-0.227	0.032	<0.0001	-0.289 to - 0.164	0.202
Acne	Acne	-1.212	0.220	<0.0001	-1.64 to -0.77	0.180
Weight	BMI	-0.787	0.142	<0.0001	- 1.067 to -0.506	0.150
Infertility						
	Infertility	-1.631	0.311	<0.0001	-2.245 to -1.018	0.182
	Interval between menstruation	-0.676	0.293	0.022	-1.254 to -0.098	
Menstruation	Interval between menstruation	-0.225	0.079	0.005	-0.381 to -0.069	0.016
Emotional						
	F/G score	-8.86	0.023	<0.0001	-0.133 to -0.044	0.093
	Interval between menstruation	-0.496	0.022	0.026	-0.933 to -0.059	

†Age, F/G score and BMI were included in the regression analysis as continuous variables and other variables were used as dummy variables. Only significant results are presented. * Subscales of MPCOSQ

## Discussion

To the best of our knowledge, this study is the first to evaluate the influence of the major clinical features of PCOS on HRQOL in patients using of MPCOSQ. In the current study, the subscales of MPCOSQ predicted well with study variables that addressed specific PCOS symptoms, supporting the validity of subscales of MPCOSQ. In this study, menstrual problems were the greatest concern reported by the PCOS women. The current study demonstrated that the patients have cycle irregularities, the lower the scores (greater concerns) reported for the menstruation subscale. 

The clinical characteristics of the sample may explain why the menstrual domain was reported to have the most negative influence on HRQOL because the majority of participants have oligomenorrhea, amenorrhea or changeable menstruation (49.5%, n=109). Infertility problems ranked the second highest of concern on the MPCOSQ and predicted by having menstrual irregularities and infertility. As noted earlier, more than half of the women in the study had infertility. 

Menstrual irregularities, i.e. no regular cycle and sometimes even no menstrual bleeding without the use of medication, are strongly related with infertility, and because is a menstrual irregularities problem for these women, it is clear that they also rate their infertility a bigger problem. However, some socio-cultural generalizations are possible: the social pressure to have a child shortly after marriage is strong in Iran. Current study findings are in general agreement with an earlier study by Winkvist *et al* (19). 

Many Islamic Pakistani women feel strongly that their childbearing pattern influences the way people treat them; they are more respected in the family when they have children. Without children, they do not feel like a real woman (19-20). In their 2003 study, Guz *et al* have reported that the reaction of the family and social group of infertile women in Turkey has an important role in the development of specific psychiatric symptoms (21). 

Although hirsutism received the third reported area of concern, it was significantly predicted by F/G score. Also, the F/G score predicted score of emotion subscale on the MPCOSQ. Hahn *et al* and McCook *et al* also reported similar findings in that the FG score correlated significantly with emotional subscale of their study instruments (2, 22). It has been suggested that women with PCOS have a lower self-esteem, a more negative self-image, higher levels of depression and psychological distress owing to the physical appearance of hyperandrogenism, including obesity, hirsutism, cystic acne, seborrhea and hair loss, possibly by influencing feminine identity (23-25). Weight and emotion received the fourth and fifth subscales reported area of concern. 

This brings into focus that menstrual irregularities and infertility perhaps plays a more important role than obesity in the occurrence of psychological distress among the Iranian women affected. This phenomenon probably reflects different socio-cultural views of obesity, which has important implications on management issues of Iranian subjects with PCOS. Schmid *et al* reported that Muslim immigrant women rated menstrual irregularity, infertility and hirsutism as being bigger problems than being overweight or obese, whereas European women perceived these quite differently. Obesity with an android pattern of body fat having a negative impact on HRQOL may be explained by it being considered unattractive only in Western cultures and modes of dress, whereas Eastern cultures might even perceive obesity as a sign of wealth (26). 

We propose that this problem needs appropriate corrective action in the clinical setting of primary care in Iranian communities, as weight reduction is an important step in the management of PCOS. Our finding also suggests the potential for non-cooperation with weight management protocols among affected Iranian women. Although, low ranking of weight concerns may be explained by the fact that only 1 out of 5 women in this study were actually overweight. However, it should be noted that it has certain limitations.

The study of patients with PCOS who attend two private gynecology clinics may limit generalization of the findings to the entire PCOS population. Also, patients referred to gynecology clinics may be different in certain socio-cultural and psychological variables compare to community. It is possible to suggest that the majority of these patients have menstrual irregularities and infertility (not obesity and acne) because these patients with other complaints are often admitted to dermatology and endocrinology clinics. 

But, the demographic findings of the present study have shown sufficient number of patient with these complaints that allow us to evaluate QOL in these patients. Moreover, all of the patients in this study were married for cultural reasons (sex and infertility) in Iran. Therefore, the results of the present study have to be interpreted with some caution. In this study, absence non-PCOS controls matched on the various clinical features prevented the assessment of their individual contributions as principal influences on QOL, separate from the diagnosis of PCOS. 

Control participants for comparison with women with PCOS are very difficult to identify in advance of screening, owing to its high prevalence and diagnostic difficulties discussed above. Comparison studies are also needed with healthy ovulatory women to better define the degree of impaired QOL resulting from PCOS.

Our findings demonstrate that reduction of HRQOL is related to more menstrual irregularities and infertility than to obesity, which is distinct from Western findings, and suggests a potential for poorer cooperation with weight management protocols among affected Iranian women. Therefore, health professionals in this field should be encouraged to be sensitive to cultural background of their patients. 

As a result, a multidimensional and cultural-based approach through health education and counseling is recommended. Moreover, we recommend a conducting qualitative research to explore variables related to poor HRQOL in these patients in Iran. Future researches should study these associations further against control populations. We did notice a trend toward a difference and a larger study may have had the power to detect relationships.
